# *Aedes aegypti* container preference for oviposition and its possible implications for dengue vector surveillance in Delhi, India

**DOI:** 10.4178/epih.e2023073

**Published:** 2023-08-23

**Authors:** Pooja Prasad, Suman Lata, Sanjeev Kumar Gupta, Pawan Kumar, Rekha Saxena, Deepak Kumar Arya, Himmat Singh

**Affiliations:** 1 ICMR-National Institute of Malaria Research, New Delhi, India; 2 Department of Zoology, D.S.B Campus, Kumaun University, Nainital, India; 3 Academy of Scientific and Innovative Research (AcSIR), New Delhi, India

**Keywords:** *Aedes*, Oviposition, Clay container, Ovitraps, Dengue

## Abstract

**OBJECTIVES:**

Dengue is a mosquito-borne viral disease globally transmitted by *Aedes aegypti*. The most effective method to prevent the transmission of the disease is proficient vector control. Understanding the breeding behaviour of the responsible vectors is very pertinent in this regard; therefore, the present study was conducted to understand *Ae. aegypti* behaviour regarding the selection of containers for oviposition in the megacity of Delhi.

**METHODS:**

A household survey in different localities within Delhi was carried out during 2018-2019. All available containers were inspected for the presence of immature *Ae. aegypti*. In entomological surveillance, the ovipositional preference of *Aedes* was computed using the breeding preference ratio, container index in the field, and laboratory settings, and associations of dengue cases with monthly variation in environmental factors and container type were also calculated.

**RESULTS:**

The household larval survey in 40 localities showed that 40% of 27,776 water-holding containers in 3,400 houses were plastic, followed by overhead tanks (26.2%), and coolers (12.1%). The most preferred breeding habitat was clay pots (9.3%), followed by metallic containers (8.5%) and solid waste (7.1%). A laboratory-based study showed that *Aedes* preferred clay containers (81.8%) over 4 other types of containers (plastic, paper, metal, and glass).

**CONCLUSIONS:**

The present study provides a rationale for using clay containers as a possible surveillance tool (ovitraps) or as a vector control tool. This information might aid researchers in developing novel traps and targeting preferred containers for larval control activities during transmission and non-transmission seasons.

## GRAPHICAL ABSTRACT

[Fig f6-epih-45-e2023073]

## INTRODUCTION

Dengue is a major global public health concern. Cases have increased worldwide more than 8-fold, from about 0.5 million cases in 2000 to over 2.4 million cases in 2010 and then 4.2 million cases in 2019 [1]. Currently, 129 countries are at risk for dengue infection [2], and 70% of the actual burden is in Asia [3-5]. In India, the *Aedes aegypti* mosquito, due to its high affinity for humans, is the most effective vector for transmitting arboviruses [6]. In 2020, India reported 39,419 dengue cases and 56 deaths, with Delhi alone accounting for 1,269 cases, albeit with no fatalities [7]. Since 1967, Delhi has experienced several dengue epidemics, with some of the most significant outbreaks occurring in 1996, 2003, 2006, and 2015 [8-12]. The high incidence of dengue cases in Delhi can be attributed to various factors. These include large population size, haphazard urbanisation, socioeconomic conditions, and changing climatic conditions. Poor water storage practices have also contributed to an increase in breeding habitats for *Ae. aegypti* [13-16]. In the absence of a commercially available vaccine for dengue, controlling the vector remains the most effective strategy to curb its transmission. The key to managing dengue lies in adopting a comprehensive approach. This includes ongoing vector surveillance, the integrated management of *Aedes* mosquitoes using safe and cost-effective biological and chemical controls, environmental management, legislation, and action at both individual and community levels.

*Ae. aegypti*, often referred to as a container breeder mosquito, typically breeds in domestic and peri-domestic man-made containers such as plastic drums, overhead tanks, cans, and buckets, as well as natural containers containing some organic matter, like tree holes [17,18]. The wide variety of water containers available for egg-laying makes it challenging to control the *Ae. aegypti* population. Therefore, studies on breeding site preferences are crucial for planning surveillance and monitoring within vector control programs. A study conducted by Vikram et al. [19] between 2013 and 2014 in 18 Delhi localities with varying socioeconomic groups found that containers in low-income areas contributed more to *Ae. aegypti* breeding than those in middle-income and high-income localities. Given that the ecological parameters of this species can change over time and space, it is necessary to have updated information to plan and implement effective control measures against the *Ae. aegypti* mosquito in Delhi. Consequently, this study aimed to provide insight into the preferred breeding containers of *Ae. aegypti* among the commonly available containers in the community.

## MATERIALS AND METHODS

### Study site

The research was conducted in Delhi, the capital of India, from September 2018 to December 2019. Delhi is a metropolitan city that spans an area of 1,483 square kilometres. It is situated at the intersection of 28.53º north latitude and 77.20º east longitude. The city is nestled on both sides of the Arawali Hill range and is bordered on 3 sides by Haryana, while Uttar Pradesh lies across the river, Yamuna. Delhi is governed by 3 civic bodies: the East Delhi Municipal Corporation, the North Delhi Municipal Corporation, and the South Delhi Municipal Corporation. These bodies have been unified into a single entity, the Municipal Corporation of Delhi, which is divided into 12 zones. The study was divided into 2 components (fieldwork and laboratory work), to determine the oviposition preference of *Ae. aegypti*.

### Field study

**Figure 1.**
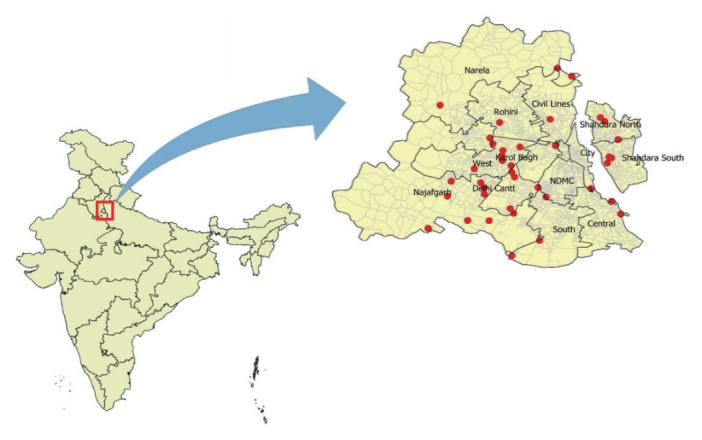
Surveyed localities (40 localities) within Delhi.

We conducted monthly entomological surveys in 40 localities within different zones of Delhi (Figure 1). The localities were selected based on the incidence of dengue in previous years, and to ensure a broad representation of Delhi’s geographical area. After the localities were selected, we visited them regularly each month. However, the specific houses within these locations were selected randomly.

**Figure 2.**
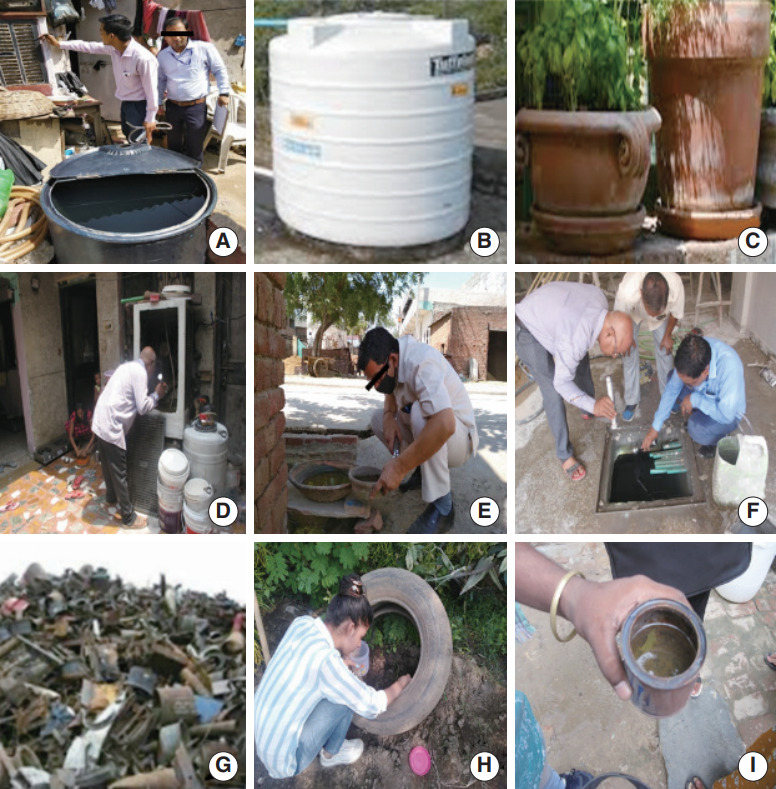
*Aedes aegypti* mosquito breeding habitats identified during entomological surveillance within the metropolitan city of Delhi, India: (A) plastic container, (B) overhead tank, (C) flower pot, (D) cooler, (E) clay pot, (F) cemented tank, (G) solid waste, (H) discarded tires, and (I) metal container.

The household surveys were planned with Municipal Corporation of Delhi workers who conducted door-to-door inspections for larvae. In every household, we examined water-holding containers, including overhead tanks, cement tanks, underground tanks, metal and plastic containers, bird pots, flowerpots, discarded tires, and solid waste (such as unused bottles, coconut shells, and rotten leaves) for any life stage of the *Ae. aegypti* mosquito (Figure 2).

The containers were labelled as inspected if they contained water, and marked as positive if they housed any immature forms, larvae, or pupae. Depending on the type of container, mosquito samples were gathered using either a pipette or a dipper. Larvae were collected from their natural habitats and then reared in the laboratory until they matured. Following their emergence, they were identified using standard keys.

### Laboratory study

**Figure 3.**
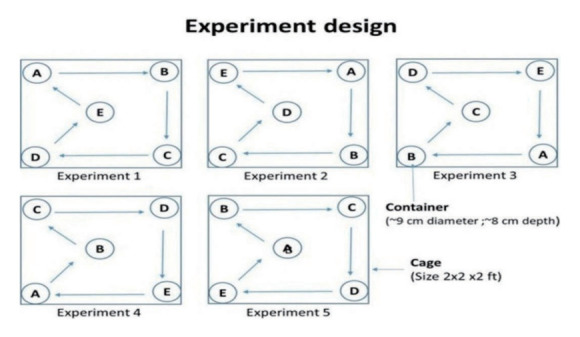
Experimental design for the placement of breeding container types (A: clay, B: plastic, C: paper, D: metal, and E: glass) in cages.

The laboratory experiments took place at the ICMR-National Institute of Malaria Research in Delhi. These experiments were carried out in pairs, independently, within cages measuring 2× 2× 2 ft. In order to determine the egg-laying preference of *Ae. aegypti*, we placed 250 mL water cups in a cage, 1 for each of the 5 containers. These containers, all of the same size, were made from different materials: clay, plastic, paper, metal, and glass. Each container had a diameter of approximately 9 cm and a depth of 8 cm (Figure 3).

Dark-coloured containers were utilised for this experiment, including black plastic, dark paper, and red-brown clay containers, while the glass containers were transparent. There were 5 replications per container type, and for each replication, 10 gravid *Ae. aegypti* females were introduced into 2 cages (cages 1 and 2) and allowed to lay eggs within the containers. The laboratory conditions were maintained at a temperature of 25± 2°C and relative humidity between 65% and 70%. After 4 days, the containers were removed from the cages and inspected for the presence of eggs. The eggs were counted using a hand lens, and the water in each container was examined for additional eggs or hatched larvae. For each replication, a fresh set of containers was used, rotated clockwise from their previous positions. The total number of eggs was then tallied for analysis. The breeding preference was determined by the number of eggs deposited in each container within the laboratory setting and was calculated using the percentage of egg laying.

### Statistical analysis

Dengue incidence data were collected from the DMC, and meteorological data (temperature and rainfall) were obtained from the National Aeronautics and Space Administration Langley Research Center Prediction of Worldwide Energy Resource Project (funded through the NASA Earth Science/Applied Science Program and Ministry of Earth Science, India Meteorological Department; released under the National Data Sharing and Accessibility).

To determine the percentage of water-holding containers that were infested with mosquito larvae or pupae, the container index (CI) was calculated using the following formula:


$$Container\;index\;(CI)\;=\;\frac{Number\;of\;positive\;containers}{Total\;number\;of\;containers\;checked}\times 100$$

To measure the attractiveness of different stimuli, such as the colour or odour of a particular stimulus, for *Ae. aegypti* female mosquitoes, the breeding preference ratio (BPR) was estimated using the following formula:


$$\mathrm{Breeding}\;\mathrm{preference}\;\mathrm{ratio}\;(\mathrm{BPR})\;=\;\frac{Y}{X}$$

Where,


$$X(\%)=\frac{Number\;of\;examined\;water\;reservoir\;\mathrm{container}s\;for\;each\;type}{Total\;number\;of\;examined\;water\;reservoir\;\mathrm{container}s}\times 100\;Y(\%)=\frac{Number\;of\;larvae-positive\;water\;reservoir\;\mathrm{container}s\;for\;each\;type}{Total\;number\;of\;larvae-\mathrm{positive}\;\mathrm{water}\;reservoir\;\mathrm{container}s}\times 100$$

The Pearson correlation coefficient between environmental factors and dengue cases was calculated utilising dengue cases and meteorological data from different sites. For the laboratory experiments, the chi-square (*χ*^2^) significance test was applied to ascertain the preferred behaviour of the *Ae. aegypti* mosquito for each type of container.

### Ethics statement

The study protocol was approved by the Research Integrity Committee (RIC) of Institute 12/2021.

## RESULTS

### Field study

About 3,400 houses in 40 localities (80-90 houses/locality) in Delhi were surveyed from July 2018 to August 2019 for the presence of *Ae. aegypti* life stages in various water-holding containers. These containers varied in number, type, and size. The localities were selected randomly but stratified based on the incidence of dengue.

In total, 26,776 containers were checked, and 2.1% (579 containers) were found positive for *Ae. aegypti* breeding. A container was deemed positive if it housed even a single immature life stage of the mosquito species (Table 1). The current study found that 97.7% of the inspected containers were overhead tanks, flowerpots, coolers, and plastic containers. In an urban environment, the majority of water storage containers, such as drums, buckets, and overhead tanks, are composed of plastic (67%).

Plastic water storage containers (40.7%) and overhead tanks (26.2%) were the most common in the community, contributing positively at rates of 37.8% and 27.5%, respectively. Coolers were also frequently found (12.1%), contributing positively at a rate of 25.0%. Clay containers were less common (1.1%), but their positive contribution was still notable at 4.8% (Table 1). Throughout the entire study period, only 3 underground cement tanks were discovered. Due to the disproportionate sample size of underground tanks compared to other containers, data on cement tanks and underground cement tanks were combined. Apart from flowerpots (0.30%), the percentage of water-holding containers infested with larvae or pupae, known as the container index, varied from 2.01% (plastic containers) to 9.36% (clay containers). The highest breeding preference ratio was found in clay pots (4.33), followed by metal containers (3.94), solid waste (3.30), and coolers (2.07). The container positivity among solid waste, tires, and metallic containers was observed to be less than 1% and therefore could be considered negligible.

### Laboratory-based study

In a controlled laboratory environment, 100 gravid females laid a total of 3,524 eggs. These females were divided into groups of 10 and housed in separate experimental cages. The female *Ae. aegypti* mosquitoes showed a significant preference for clay containers, with a notably high egg-laying rate of 81.8% compared to containers made from other materials such as plastic, paper, metal, and glass (Table 2).

The study found that *Ae. aegypti* showed a strong preference for clay containers (81.8%), irrespective of their location within the cage. This was followed by plastic containers (11.1%), paper (2.5%), and metallic containers (4.2%). Glass containers were the least favoured, with a preference rate of only 0.5%. The chi-square test indicated a significant difference in the number of eggs laid by *Ae. aegypti* depending on the type of container used (*χ*^2^ = 3,924.139; df=4; p<0.01).

**Figure 4.**
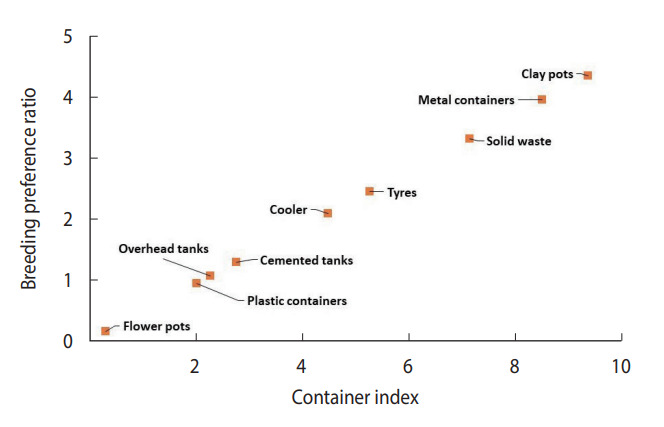
Preference of female *Aedes aegypti* for containers in natural field conditions.

**Figure 5.**
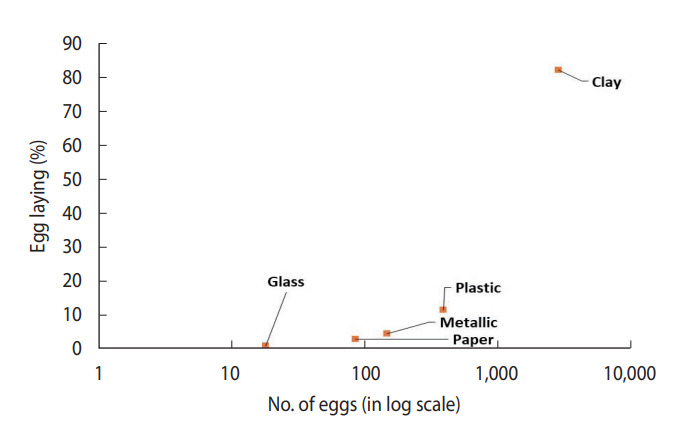
Percent egg laying by female *Aedes aegypti* in containers in laboratory conditions (the number of eggs on the x-axis is in log scale).

The scatterplot between CI and BPR for field conditions revealed that clay containers were the most preferred containers, whereas flowerpots were the least preferred containers in terms of both CI and BPR (Figure 4). In laboratory settings, *Ae. aegypti* also preferred to breed in clay containers (Figure 5) over other available containers.

### Seasonal and environmental dynamics

As shown in Supplementary Material 1A, July was the month with the most rainfall, followed by August. In contrast, Supplementary Material 1B indicates that the container index peaked in August, then September, suggesting heightened mosquito activity during these months. Additionally, the most significant number of dengue cases were reported between October and November (Supplementary Material 1B). This pattern can be explained by the time lag that occurs from when mosquitoes lay their eggs after rainfall, through the hatching and maturation process, to when they begin feeding on the blood of dengue fever patients. It takes time for the virus to migrate from the mosquito’s midgut cells to its salivary glands. This extrinsic incubation period is also influenced by factors such as ambient temperature and the mosquito’s competence. Once the dengue virus has reached the salivary gland of an infected mosquito, it can be transmitted to another person through a subsequent blood meal. This person then goes through an incubation period before symptoms appear. The current study found a significant positive correlation (r=-0.38, p<0.05) between the average temperature increase from July to November and the number of dengue cases (Supplementary Material 2). The increase in the number of breeding containers is likely to correspond to a rise in dengue cases. Therefore, the results of this study imply that initiatives aimed at reducing breeding containers could potentially contribute to reducing the incidence of dengue cases.

## DISCUSSION

Vector surveillance is an important tool for generating entomological data necessary for effective *Ae. aegypti* control strategies, and understanding the breeding site selection of vector mosquitoes is a vital component of this process. *Ae. aegypti* mosquitoes breed indiscriminately in several types of habitats, and their oviposition among different habitats is influenced by several factors, such as the size and type of container [21-23], the presence of conspecific larvae and pupae (semiochemicals) [24], and sun exposure [25]. Studies have demonstrated that the colour and material of containers were important determinants for container preference by *Ae. aegypti* mosquitoes and affected *Ae. aegypti* egg-laying preferences [26,27]. Colour adaptations are helpful in insect survivorship and species fitness [28,29]. The colour preference of *Ae. aegypti* females is primarily based on a greater attraction to dark surfaces [30], suggesting that *Ae. aegypti* prefer dark breeding areas, over other colours such as blue, yellow, and white [31]. Colton et al. [32] reported that *Aedes* mosquitoes lay the majority of their eggs in black ovitraps, possibly to protect the eggs and offspring from predators, as the eggs are typically black. A study by Harrington et al. [33] found that *Ae. aegypti* shows a positive correlation between increasing egg numbers and increasing container volume, indicating that females are under selection pressure to choose a container that will provide the highest offspring survival rates.

Delhi, a densely populated city with an inadequate water supply and a diverse mix of residents from various socioeconomic levels and housing types [34,35], faces unique challenges. The lack of access to tap water leads to an increase in water-bearing container storage in homes, which can become breeding grounds for mosquitoes due to the storage of clean water in jars. It has been observed that the most deprived areas also have the poorest water access and the highest proportion of containers positive for mosquitoes [19]. Socioeconomic factors such as intermittent water supply, leading to more water storage in containers without proper lids, improper solid waste management, and lack of civic amenities, all contribute to the breeding of *Ae. aegypti*. In the case of dengue vectors, a sudden increase in density after rainfall could be due to the emergence of many larvae as a result of the massive hatching of eggs accumulated on container walls during a dry period. Various studies have shown that the primary breeding containers may change according to the availability of container types in an area [36,37]. One study demonstrated that *Ae. aegypti* preferred to breed on the vertical walls of a container, which was the main reason for the high productivity of cement containers [38]. Our findings are also supported by Bisht et al. [39] and might explain why the present study found a sharp peak in October after a lag phase of a month for dengue cases. A similar pattern with a lag phase from 1 to 3 months was observed in Cambodia [40], Brazil [41], and Townsville [42,43].

In this study, clay containers were the most favoured for egg-laying, with a preference rate of 81.8%, regardless of their location. This finding aligns with a study by Basra et al. [34] conducted in the Shahdara zone of Delhi, which found earthen pots to be the most productive breeding container under field conditions. This preference may be due to the porous, rough surfaces of clay pots, which are more conducive to breeding than plastic and iron containers. The *Aedes* mosquito’s affinity for clay pots suggests that it practices “skip” oviposition, laying eggs in other available containers, such as plastic ones, when clay pots are not available. Despite offering suitable conditions for breeding, cement tanks and tires were not identified as major contributors to *Aedes* breeding in this study. This could be due to the application of the insecticide temephos to these unattended containers by domestic breeding checker workers in Delhi. Historically, *Ae. aegypti* was known to breed in indoor water storage containers. However, recent studies have indicated that *Ae. aegypti* may have developed a new adaptive strategy to breed outdoors. This shift could be attributed to several factors, including urbanisation, climate change, and insecticide resistance [19,44,45]. In the context of Delhi, India, bird pots placed on rooftops could potentially serve as breeding sites for *Ae. aegypti*. These pots can collect rainwater, providing a suitable breeding habitat for mosquitoes. If not cleaned regularly, they can also accumulate organic matter, which can serve as a food source for mosquito larvae. It’s important to note that this shift towards outdoor breeding by *Ae. aegypti* could significantly impact mosquito control strategies. Traditional indoor-based control measures, such as insecticide-treated nets and indoor residual spraying, may not be effective against mosquitoes that breed outdoors.

The suppression of *Ae. aegypti* is a practical method for controlling urban dengue, yellow fever, and chikungunya viruses. Over the past 50 years, many methods have relied on source reduction, but this approach appears to be ineffective due to insufficient surveillance to minimise the vector population and disease load. To develop more effective surveillance and control tools for outbreak detection, it is crucial to understand the current behaviour of the vector species. As such, there is a need for new control measures that target the outdoor breeding habitats of mosquitoes.

Despite the main limitation of this study, which was the varying sizes of household containers surveyed, from large (overhead tanks) to small (plastic bottles), we conclude that clay containers could be used in the future to attract the *Aedes* mosquito for oviposition. Targeting the preferred containers could be a cost-effective way to reduce the vector population and arboviral transmission in a given area or region. These results demonstrate that clay pots were the most preferred containers and could potentially be used as a surveillance tool (ovitraps) or as a vector control tool for the elimination of arboviral infections.

## Figures and Tables

**Figure f6-epih-45-e2023073:**
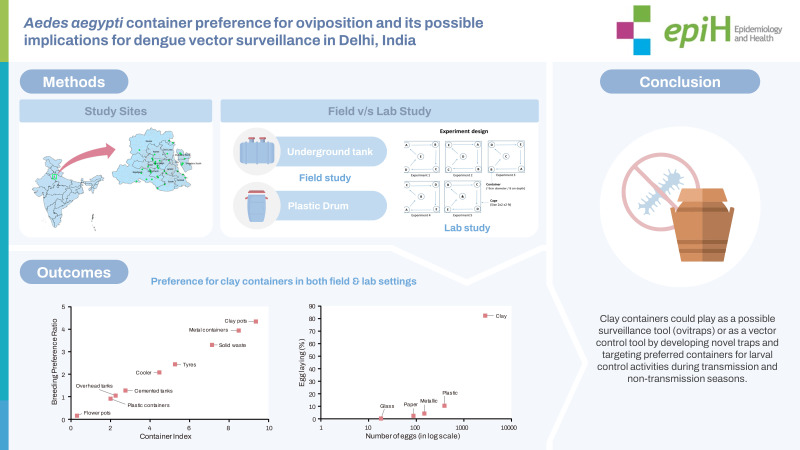


**Table 1.** Container index and breeding preference ratio (BPR)^1^ of *Aedes aegypti* according to habitat

Breeding habitat	Container				Container index	BPR (Y/X)
	Checked (n)	Percent contribution (X)	Positive (n)	Percent contribution to positive results (Y)		
Plastic containers	10,898	40.7	219	37.8	2.01	0.93
Overhead tanks	7,013	26.2	159	27.5	2.27	1.05
Flowerpots	5,015	18.7	15	2.6	0.30	0.14
Coolers	3,240	12.1	145	25.0	4.48	2.07
Clay pots	299	1.1	28	4.8	9.36	4.33
Cemented tanks	217	0.8	6	1.0	2.76	1.28
Solid waste	28	0.1	2	0.3	7.14	3.30
Tyres	19	0.1	1	0.2	5.26	2.43
Metal containers	47	0.2	4	0.7	8.51	3.94
Total	26,776	-	579	-	2.16	-

1 BPR values less than 1 indicate that this category was unattractive to female mosquitoes and they preferred the other container stimuli, while values greater than 1 indicate that the container stimulus was more attractive than the other containers [20].

**Table 2.** Eggs laid by *Aedes aegypti* in different container types in laboratory conditions (sum of 5 replicates)

Experiment	Clay	Plastic	Paper	Metallic	Glass	Total eggs laid
Cage 1^1^	1,497	138	48	78	10	1,771
Cage 2^1^	1,386	251	38	70	8	1,753
Grand total	2,883	389	86	148	18	3,524
Egg laying (%)	81.8	11.1	2.5	4.2	0.5	-

1 Sum of 5 replicates in each cage.

## SUPPLEMENTARY MATERIALS

Supplement Material 1. *Ae. aegypti* mosquito dynamics with environmental factors. (A) Monthly temperature and cumulative rainfall in Delhi. (B) Monthly container index and dengue cases in Delhi.

Supplement Material 2. Association of dengue cases with monthly variation in environmental factors and container type
