# Supplementary material for: Aedes aegypti container preference for oviposition and its possible implications for dengue vector surveillance in Delhi, India
Source: Epidemiol Health. 2023 Aug 23;45:e2023073. doi: 10.4178/epih.e2023073 (PMC10728616; doi:10.4178/epih.e2023073)
Supplement: Supplement Material 1. — Ae. aegypti mosquito dynamics with environmental factors. (A) Monthly temperature and cumulative rainfall in Delhi. (B) Monthly container index and dengue cases in Delhi. [file epih-45-e2023073-Supplementary-1.docx]

**Supplementary material**

**(1B)**

**(1A)**

**Supplementary Material 1. *Ae. aegypti* mosquito dynamics with environmental factors. (A) Monthly temperature and cumulative rainfall in Delhi. (B) Monthly container index and dengue cases in Delhi.**
