# Supplementary material for: Aedes aegypti container preference for oviposition and its possible implications for dengue vector surveillance in Delhi, India
Source: Epidemiol Health. 2023 Aug 23;45:e2023073. doi: 10.4178/epih.e2023073 (PMC10728616; doi:10.4178/epih.e2023073)
Supplement: Supplement Material 2. — Association of dengue cases with monthly variation in environmental factors and container type [file epih-45-e2023073-Supplementary-2.docx]

**Supplementary Material 2. Association of dengue cases with monthly variation in environmental factors and container type**

| **Months** | **Temperature (°C)** | **Cumulative rainfall (mm)** | **Total checked containers** | **Positive container** | **Container index (CI)** | **Dengue cases** |
| --- | --- | --- | --- | --- | --- | --- |
| Sep-18 | 26.97 | 790.4 | 2205 | 30 | 1.36 | 374 |
| Oct-18 | 24.23 | 790.4 | 2242 | 35 | 1.56 | 1114 |
| Nov-18 | 19.74 | 793.9 | 2462 | 34 | 1.38 | 1062 |
| Dec-18 | 13.8 | 794.4 | 1743 | 22 | 1.26 | 141 |
| Jan-19 | 12.65 | 35.7 | 697 | 3 | 0.43 | 1 |
| Feb-19 | 15.26 | 71.1 | 1013 | 9 | 0.89 | 1 |
| Mar-19 | 21.15 | 76.2 | 2027 | 40 | 1.97 | 4 |
| Apr-19 | 30.72 | 89.9 | 1114 | 9 | 0.81 | 2 |
| May-19 | 33.61 | 114 | 1481 | 20 | 1.35 | 3 |
| Jun-19 | 37.48 | 117.3 | 971 | 32 | 3.3 | 11 |
| Jul-19 | 33.55 | 261.1 | 1628 | 45 | 2.76 | 18 |
| Aug-19 | 30.25 | 399.9 | 1658 | 100 | 6.03 | 52 |
| Sep-19 | 29.65 | 448.2 | 1715 | 80 | 4.66 | 190 |
| Oct-19 | 25.45 | 464.4 | 1416 | 54 | 3.81 | 787 |
| Nov-19 | 20.98 | 472 | 680 | 22 | 3.24 | 717 |
| Dec-19 | 12.57 | 514.7 | 1315 | 16 | 1.22 | 250 |
